# Therapeutic Effect of Epigallocatechin-3-gallate (EGCG) and Silibinin on ATM Dynamics in Prostate Cancer Cell Line LNCaP

**DOI:** 10.4021/wjon248w

**Published:** 2011-01-01

**Authors:** Ammad Ahmad Farooqi, Qaisar Mansoor, Muhammad Ismail, Shahzad Bhatti

**Affiliations:** a Institute of Molecular Biology and Biotechnology (IMBB), The University of Lahore, Pakistan; b Institute of Biomedical and Genetic Engineering (IBGE), Islamabad, Pakistan

**Keywords:** Epigallocatechin-3-gallate (EGCG), Silibinin, Prostate cancer, LNCaP cell line

## Abstract

**Background:**

Epigallocatechin-3-gallate (EGCG) is a major ingredient of green tea (GT) and silibinin (SB), the active component of Silymarin presumably hold a potential to prevent pathogenomics. Prostate cancer exacerbation is triggered by fusion transcripts formed because of genomic instability induced by juxtapositioning of two genes. This chimeric transcript is implicated in androgen dependent and independent prostate cancer. Tremendous work is done on the characterization of the mediators involved in the disease refractoriness, yet no study has addressed clinical management of these prostate fusion transcripts impressively.

**Methods:**

An abolished ATM dynamics challenges integrity of DNA. In agreement with this assumption, ATM and DNA-PK were impaired in LNCaP cell line to confirm a tight interaction of these mediators with the expression profile of TMPRSS2-ERG. Abolished ATM enhanced the expression of the fusion transcript. Similarly blunting of DNA-PK downregulated the expression of the fusion transcript giving a notion that DNA-PK is involved in the chromosomal translocation. LNCaP cell lines were analyzed for the effect of EGCG and SB on the expression profile of TMPRSS2-ERG.

**Results:**

In this particular unprecedented study, treatment of the LNCaP cell line with EGCG and Silibilin recapitulated ATM expression and activity and downregulated the fusion transcript appearance.

**Conclusions:**

These results underscore the therapeutic effect of EGCG and SB in mitigating the exacerbation of the disease with reference to the fusion transcripts.

## Introduction

Prostate cancer is modulated by fusion transcripts. These chimeric transcripts were most common in hematological malignancies. However, few years back, these were reported in prostate epithelium. The generation of these transcripts is a result of genome rearrangements. These alterations or rearrangements disturb the individuality of the gene and there is a partial sharing between two incomplete genes to carry on the illegitimate dynamics of the neoplastic cell. The chromosomal translocation or genomic rearrangements are generated because of unfaithful repair of the genome after DNA damage. The demolition of genomic stability leads to oncogenesis. TMPRSS2-ERG is a fusion transcript that is a well documented example of genomics instability outcomes in prostate epithelium [1, 2]. Tomlins identified recurrent gene fusions of the 5' untranslated region of TMPRSS2 to ERG, ETV1 or ETV4 in prostate cancer tissues with outlier expression. Although TMPRSS2-ERG fusions are predominant, fewer TMPRSS2-ETV1 and ETV4 cases have been identified on the basis of the frequency of high (outlier) expressions respectively [1-3]. According to Chunru et al [4], when androgen binds to the androgen receptor, this leads to chromosomal translocations. There is a collaboration of AR and some other proteins that work in conjunction to induce genomic rearrangements. TOP2B, AID and GADD undergo co-recruitment with AR for genomic rearrangements [5]. Androgen signaling facilitates the 5 and 3 gene fusion partners, thereby increasing the probability of a gene fusion when subjected to agents that cause DNA double-strand to break. Similarly the mutant DNA repair genes were proposed to be susceptibility genes for TMPRSS2-ERG fusion-positive PCa [6, 7]. A number of naturally derived food substances have been studied in prostate cancer in an attempt to identify natural preventative therapies for this disease. Epigallocatechin-3-gallate (EGCG) either alone or in combination can be a useful tool in the clinical management of prostate cancer [8-10]. Similarly silibinin (SB) has some remarkable results in the treatment of the disease [11-13].

## Materials and Methods

### Cell lines and treatments

The prostate cancer cell line LNCaP was obtained from the American Type Culture Collection. Human PrECs was maintained in PrEGM media. Transfection of LNCaP and PrECs cells with siRNA was performed with Lipofectamine2000 (Invitrogen). For induction of chromosomal translocation, LNCaP cells or PrECs were grown in charcoal-stripped serum containing media for 48 h followed by mock, DHT (10–7 M), g-irradiation (50 Gy) treatment, or both. After treatment, the cells were reincubated for 24 h before being harvested for appropriate assays. Cells were then treated with 100 µM EGCG for indicated times. Cell viability in cultures before and after irradiation was assessed by trypan blue exclusion.

### RNA interference

The ATM siRNA (sc-29761) and siRNA DNA PK (sc-35200) target sequences generally consisted of pools of three to five target-specific 19-25 nt siRNAs designed to knockdown gene expression and were purchased from Santa Cruz biotechnology.

### Western blot analysis

LNCaP cells were treated with designated concentrations of SM or SB in the presence of 1 nM Mib for 24 h. Then cells were harvested and whole cell lysate and nuclear extract were prepared and Western blotting analysis was performed as described previously by Wen et al, 2001 [14]. The suitable control antibody for ATM (sc-23922) and DNA PK (sc-5282) were purchased from Santa Cruz biotechnology.

## Result

**Figure 1:**
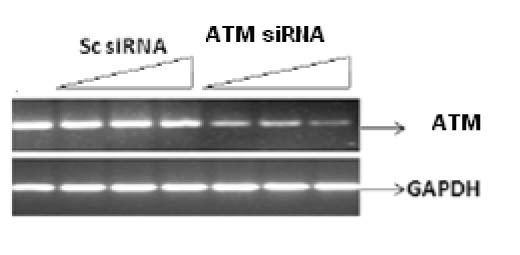
Silencing of ATM gene at transcriptional level by their specific RNA in a dose dependant manner. Cell line was infected with 25 nM, 50 nM and 100 nM of siRNA against ATM for 48 h. Scrambled RNA was used indicating non-specific targeting. Results were analyzed by reverse transcriptase PCR.

**Figure 2:**
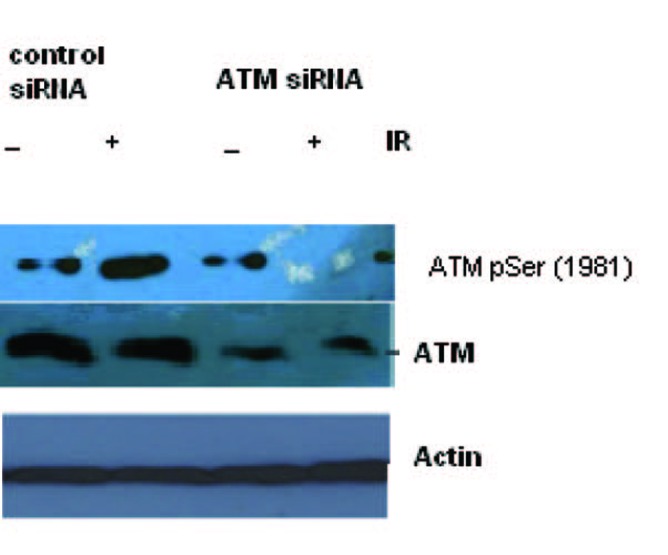
Total cell lysate was transduced with siRNA ATM and used for Western blot analysis for ATM. The blotting shows the appearance and activation of the ATM protein after irradiation in Scrambled RNA treated cells but not in the SiRNA treated cells.

Figure 1 shows the specific targeting and silencing of ATM by siRNA. In Figure 2, ATM is evaluated at protein level after treatment of the cells with siRNA. The main purpose was to identify the proficient role of ATM in the DNA repair. ATM is inactive in its unphosphorylated state. We used scrambled RNA and specific RNA for ATM to specify the role of activated ATM in DNA repair. The cells infected with ATM siRNA were unable to initiate a DNA damage response after irradiation because ATM protein was abolished and there was no autophosphorylation at serine residue 1981. Contrarily, the cells infected with scrambled siRNA displayed the expression of ATM and its activation after irradiation. Altogether the results indicate that ATM activation (autophosphorylation) is primarily dependent upon DNA damage.

**Figure 3:**
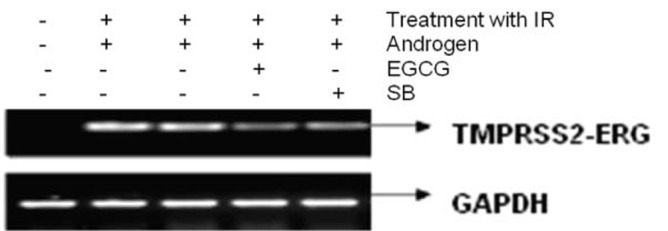
The figure shows the attenuation of expression of the fusion transcript in LNCaP cell line after treatment with EGCG and SB.

**Figure 4:**
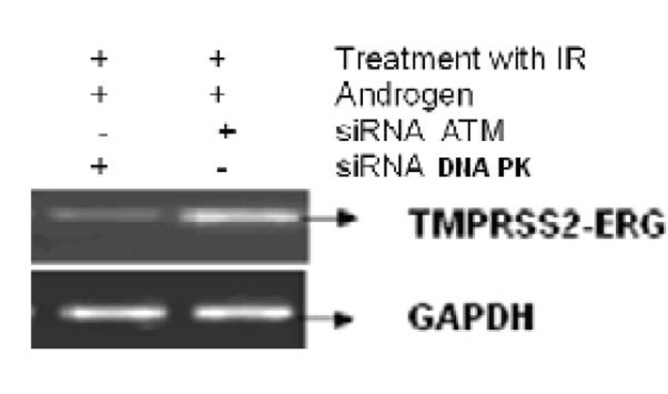
The figure shows the correlation of ATM and DNA-PK in generation of fusion transcripts. LNCaP cell line was treated with siRNA against ATM and DNA-PK successively.

**Figure 5:**
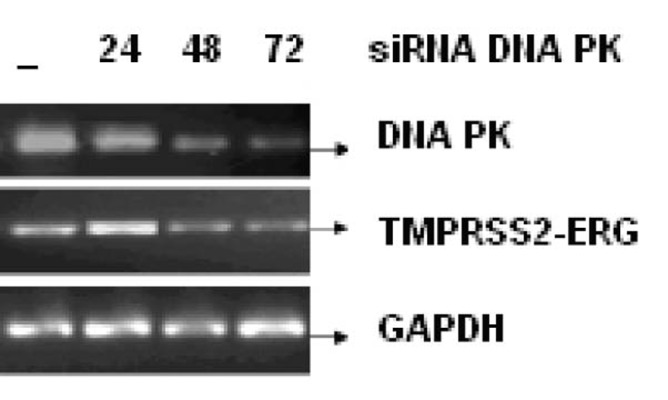
LNCaP cell line was infected with or without 25 nM, 50 nM and 100 nM of siRNA against DNA PK for 24, 48 and 72 h respectively. The mRNA transcript of fusion gene and DNA-PK was reverse transcribed. GAPDH was used as a control.

**Figure 6:**
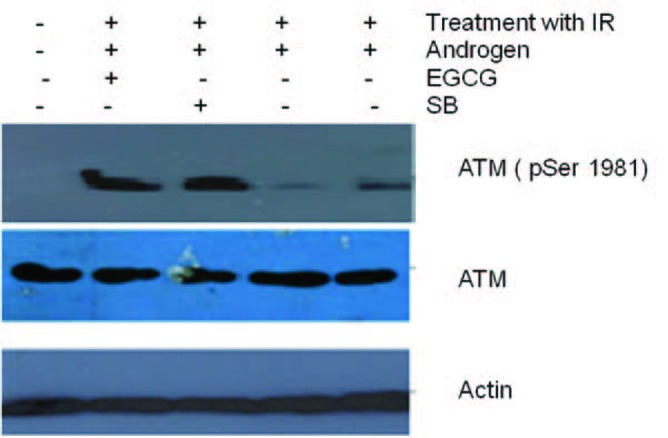
Total cell lysates from cells were irradiated and pre-treated with androgen. Later the cells treated with 100 µM EGCG for 48 and 96 h were subjected to Western blotting for ATM and their activation respectively. Actin is used as a control. Figure shows an ablation of TMPRSS2-ERG after treatment of cell line with DHT and radiation. However, post radiation treatment of Cell line with EGCG and SB stimulated ATM phosphorylation.

Figure 3 shows the effect of EGCG and SB on the fusion transcript generation in prostate cancer cell line. It was documented by Chunru et al [4] that irradiation and treatment of the LNCaP cell line with androgen stimulated the expression of TMPRSS2-ERG fusion transcripts. The enhanced genomic rearrangement was a result of the compromised activities of ATM. That was evaluated in the Figure 4 where ATM impaired cells were susceptible to genomic rearrangements. On the other hand, genomic rearrangement was promoted by DNA-PK. We observed a down regulation of the fusion transcripts after compromising DNA-PK with siRNA (Fig. 5). In one frame of experimentation we treated the cell line with siRNA of ATM and DNA-PK individually to identify the potential role of the mediators in the fusion transcript generation. We came up with the observation that ATM suppresses while DNA-PK promotes fusion transcript generation. In the other framework of experimentations we showed that SB and EGCG were competent to suppress the fusion transcript generation. In Figure 6, LNCaP cell line pretreated with androgen, and irradiation underwent attenuation of fusion transcript generation after treatment with SB and EGCG. The genomic stability was maintained by the activation of ATM which repaired the genome faithfully.

## Discussion

Since the discovery of the fusion transcripts in prostate cancer, tremendous work has been done on the characterization of novel chimeric transcripts and the underlying mechanism. In most cases, the disease progresses to castration resistant prostate cancer (CRPC). However, therapeutic interventions are ignored in this time span. Only recently two studies were conducted by Palanisamy et al, 2010 and Pfeiffer et al, 2010 respectively. Palanisamy et al [15] worked on the therapeutic intercessions for SLC45A3-BRAF (solute carrier family 45, member 3-v-raf murine sarcoma viral oncogene homolog B1) and ESRP1-RAF1 (epithelial splicing regulatory protein-1-v-raf-1 murine leukemia viral oncogene homolog-1) gene fusions. They stated that expression of SLC45A3-BRAF or ESRP1-RAF1 in prostate cells induced a neoplastic phenotype that was sensitive to RAF and mitogen-activated protein kinase (MAP2K1) inhibitors.

Pfeiffer et al [16] used an antiandrogen (bicalutamide) and a histone deacetylase (HDAC) inhibitor to induce apoptosis in TMPRSS2-ERG expressing DuCaP-N cell line. This study is still debatable as HDACi and antiandrogens are ineffective in promiscuous and outlaw driven pathway. Moreover, HDAC inhibitor-induced activation of KLK4 and NF-kappaB prevents apoptotic response [17, 18]. Additionally, induction of apoptosis is desensitized in p53 compromised cell line [19].

We have explored the role of DNA repair genes in the induction of chromosomal translocation in this particular study. Any damage done to DNA drastically compromises the activities of the cell. To resume these activities there must be a repair of the DNA. Normally homologous recombination is mediating the repair, yet any hampering of the mediators of HR results in switching to NHEJ mode. This mode facilitates the genomic rearrangements. Androgen receptor is a key player in the stimulation of chromosomal rearrangements. In prostate cancer, the activity of ATM is compromised, and repair activities are carried out by DNA-PK. This protein triggered the appearance of the fusion transcripts in prostate cancer line. Nutraceuticals are 'natural' substances isolated or purified from food substances and used in a medicinal fashion. EGCG efficiently stabilized ATM activation and that was evident from the phosphorylation of ATM. The restoration of compromised ATM dynamics in our experiments is consistent with the study conducted by Amin et al [20] who documented the ATM dependent Ser15 phosphorylation of p53 due to DNA damage after treatment with EGCG. Consistent with the same line, we were able to retrieve impaired ATM activation which is in concordance with the evidence provided by Tyagi et al [21]. They confirmed that SB mediated ATM-Chk2 pathway is essential for downstream signaling. Analogously, we were unable to obtain positive regulation of DNA-PK by SB in our list of experiments which is in contradiction with the data documented by Dhanalakshmi et al [22], who stated that SB pretreatment strongly enhanced H2A.X-Ser(139) phosphorylation and DNA-PK-associated kinase activity as well as the physical interaction of p53 with DNA-PK.

According to our interpretations, DNA-PK is actively engaged in the induction of genomic rearrangement. Furthermore, we were able to downregulate the expression of TMPRSS2-ERG in the LNCaP cell line. To date, there is no study that specifically encompasses the expression pattern of fusion genes after treatment with nutraceuticals. Our particular study demonstrates that ablation of DNA-PK and recapitulation of ATM activities are indispensable for the suppression of gene fusions. The data in this study unmasked this tight correlation of push and pull between DNA repair genes in suppression or promotion of genomic instability. Still, the pro-apoptotic potential of these two compounds remains to be investigated. Various studies have been conducted in terms of exploring the role of DNA-PK in induction of apoptosis.

One body of evidence suggests that phosphorylation of IGFBP-3 by DNA-dependent protein kinase (DNA-PK) at Ser(156) is functionally critical in its apoptosis-inducing actions. In the absence of DNA-PK activity, IGFBP-3 has reduced nuclear accumulation [23]. On the other hand, another family member of IGFBP, namely Insulin-like growth factor binding protein-2 stimulation significantly upregulated the major DNA repair enzyme gene, DNA-PKcs, and induced DNA-dependent protein kinase catalytic subunit protein expression. This robustness of expression plays a role in astrocytoma progression by promoting DNA-damage repair and therapeutic resistance [24]. Consistent with the same role in cancer progression, Miyake et al [25] registered that insulin-like growth factor (IGF) binding protein-5 (IGFBP-5) is highly upregulated in normal and malignant prostate tissues after androgen withdrawal. IGFBP-5 overexpression in prostate cancer cells after castration is an adaptive cell survival mechanism that helps potentiate the anti-apoptotic and mitogenic effects of IGF-I, thereby accelerating progression to androgen independence. The later findings were re-evaluated and blunting of DNA-PK with siRNA perhaps resensitized the cells to apoptosis. As decrease of IGFBP-2 suppressed the expression of DNA-PK, calculations obtained through flow cytometry were remarkable. Contrarily, we were unable to observe a decrease in the transcriptional firing rate of the IGFBP mediated genes after compromising DNA-PK in chromatin immunoprecipitation assay (unpublished observations).

It is believed that numerous of these 'natural' compounds have therapeutic potential and surely studies consisting of well-designed clinical trials assessing combinations of compounds concurrently will enable us to get a step closer to rational drug design. Yet a closer look into the crosstalks between proteins must be given utmost significance while trying to get a step closer in the standardization of therapy.
